# The shift in the balance between osteoblastogenesis and adipogenesis of mesenchymal stem cells mediated by glucocorticoid receptor

**DOI:** 10.1186/s13287-019-1498-0

**Published:** 2019-12-05

**Authors:** Lizhi Han, Bo Wang, Ruoyu Wang, Song Gong, Guo Chen, Weihua Xu

**Affiliations:** 1 Department of Orthopaedics, Union Hospital, Tongji Medical College, Huazhong University of Science and Technology, Wuhan, 430022 China; 2 Department of Rehabilitation, Wuhan No.1 Hospital, Wuhan Hospital of Traditional Chinese and Western Medicine, Wuhan, Hubei Province China

**Keywords:** Mesenchymal stem cells, Glucocorticoid receptor, Glucocorticoids, Osteonecrosis, Osteoporosis, Osteoblastogenesis, Adipogenesis, Stem cell therapy

## Abstract

Mesenchymal stem cells (MSCs) are multipotent cells capable of differentiating into several tissues, such as bone, cartilage, and fat. Glucocorticoids affect a variety of biological processes such as proliferation, differentiation, and apoptosis of various cell types, including osteoblasts, adipocytes, or chondrocytes. Glucocorticoids exert their function by binding to the glucocorticoid receptor (GR). Physiological concentrations of glucocorticoids stimulate osteoblast proliferation and promote osteogenic differentiation of MSCs. However, pharmacological concentrations of glucocorticoids can not only induce apoptosis of osteoblasts and osteocytes but can also reduce proliferation and inhibit the differentiation of osteoprogenitor cells. Several signaling pathways, including the Wnt, TGFβ/BMP superfamily and Notch signaling pathways, transcription factors, post-transcriptional regulators, and other regulators, regulate osteoblastogenesis and adipogenesis of MSCs mediated by GR. These signaling pathways target key transcription factors, such as Runx2 and TAZ for osteogenesis and PPARγ and C/EBPs for adipogenesis. Glucocorticoid-induced osteonecrosis and osteoporosis are caused by various factors including dysfunction of bone marrow MSCs. Transplantation of MSCs is valuable in regenerative medicine for the treatment of osteonecrosis of the femoral head, osteoporosis, osteogenesis imperfecta, and other skeletal disorders. However, the mechanism of inducing MSCs to differentiate toward the osteogenic lineage is the key to an efficient treatment. Thus, a better understanding of the molecular mechanisms behind the imbalance between GR-mediated osteoblastogenesis and adipogenesis of MSCs would not only help us to identify the pathogenic causes of glucocorticoid-induced osteonecrosis and osteoporosis but also promote future clinical applications for stem cell-based tissue engineering and regenerative medicine. Here, we primarily review the signaling mechanisms involved in adipogenesis and osteogenesis mediated by GR and discuss the factors that control the adipo-osteogenic balance.

## Introduction

Mesenchymal stem cells (MSCs) are capable of self-renewal, with the pluripotent potential to differentiate into mesenchymal tissues, including bone, cartilage, fat, muscle, tendon, and marrow stroma [1]. When cultured in vitro, MSCs have a stable phenotype and display a monolayer and can be induced to differentiate into osteoblasts, adipocytes, or chondrocytes [2]. Besides, MSCs possess immunomodulatory functions without immunogenicity that make them ideal candidates for use in the repair of tissue damage and treatment of inflammatory diseases [3]. MSCs hold great potential in regenerative medicine for the treatment of degenerative musculoskeletal conditions like osteoarthritis (OA) [4] and other clinical conditions such as osteogenesis imperfecta, osteonecrosis, and osteoporosis [5–7].

Glucocorticoids (GCs) are steroid hormones that are essential for life and play a vital role in health and disease. Endogenous GCs are primarily secreted by the adrenal glands and are involved in the modulation of the stress response and circadian rhythm. The pharmacological applications of GCs include use as potent immunosuppressant agents to treat chronic inflammatory diseases such as asthma, allergic shock, arthritis, and inflammatory bowel disease [8]. The physiological and pharmacological effects of GCs are mediated by the glucocorticoid receptor (GR) [9]. GCs act on virtually all types of cells through the GR, mediating important physiological effects via changes in gene expression and signaling. The GR is an intracellular protein that is ubiquitously expressed in almost every cell of the organism and interacts with chromatin to modulate the activity of numerous transcription factors in a cell type-specific manner.

Normal physiological levels of GCs are essential for development, metabolism, normal osteoblast differentiation, and bone formation. However, long-term exposure to GCs at pharmacological dosages is attributed to skeletal side effects, including osteoporosis, fracture, and osteonecrosis [10, 11]. Glucocorticoid-induced fractures occur in 30 to 50% of patients receiving long-term GC therapy, while osteonecrosis occurs in 9 to 40% of the patients [12, 13]. MSCs isolated from patients with steroid-induced osteonecrosis of the femoral head (SONFH) display an inherent defect in osteogenesis, which suggests that the osteonecrosis resulted from an insufficient repair mechanism [14]. Also, the in vitro culture of bone marrow-derived MSCs (BM-MSCs) from patients with osteoporosis is more likely to result in adipogenic differentiation rather than osteogenic differentiation [15]. Similar results can be found in dexamethasone (DEX)-induced osteoporotic mouse model that BM-MSCs from Dex-treated mice cultured in vitro are more likely to differentiate into adipocytes instead of osteoblasts than those from control mice [16].

The ability of the GCs to mediate a switch in the fate of MSCs has a significant impact on the skeletal system and is of clinical significance in stem cell-based tissue engineering and regenerative medicine. This review focuses on how GCs regulate the lineage commitment of MSCs through different signaling pathways, transcription factors, and post-transcriptional regulators, among other regulators. This remarkable advance in understanding molecular mechanisms behind the fate of MSCs mediated by GR will provide new insights into mitigating the adverse effects of GCs on bone and joints. Besides, the opinions presented in this review will provide new strategies for stem cell-based bone repair and regeneration in the future.

## Source and heterogeneity of MSCs

Mesenchymal stem cells (MSCs) were initially isolated from rat bone marrow (BM) and described as colony-forming fibroblasts (CFU-F) in 1976 [17]. These colony-forming cells have since been named “mesenchymal stromal cells” or “mesenchymal stem cells.” After that, other tissue-derived human MSCs have been found, such as human MSCs isolated from the adipose tissue, umbilical cord matrix, periosteum, synovium, tendon, lung, and dental pulp among others [18, 19]. Although the word “stem” has been used in numerous articles, “stemness” is a more strict term for defining MSCs with the ability to repair tissues in vivo [20]. In recent years, the term “tissue stem cells” has been replaced with “skeletal stem cells (SSCs)” as a new term for these cells [21, 22].

Heterogeneity in MSCs occurs not only in the proliferative and differentiation abilities among individual donor tissues but also among MSCs isolated from the same donor tissue. Besides, MSCs derived from different tissues or individual donors exhibit heterogeneity in marker profiles, gene expression patterns, and propensity to differentiate into particular lineages [23]. Contrary to the classical view of differentiation, the findings of transcriptome analysis in mice revealed that osteogenic and adipogenic signatures are not mutually exclusive, and specific subtypes of skeletal progenitors may co-express alternative lineage-specific genes [24]. Although BM-MSCs are widely used for stem cell therapy, these in vitro cultured cells are highly heterogeneous, with the potential to differentiate into many overlapping lineages, such as osteoblasts, chondrocytes, adipocytes, fibroblast, endothelial, and stromal cells. These promiscuous cells are likely to form a population including various types of stem cells rather than a homogeneous population of SSCs [25]. The application of single-cell analysis and lineage-tracing technologies in mice and humans revealed that SSCs could differentiate into osteoblasts, chondrocytes, and stromal cells, but not adipocytes. The report also revealed that perisinusoidal MSCs could give rise to adipocytes [25, 26].

## Glucocorticoid receptor

**Fig. 1:**
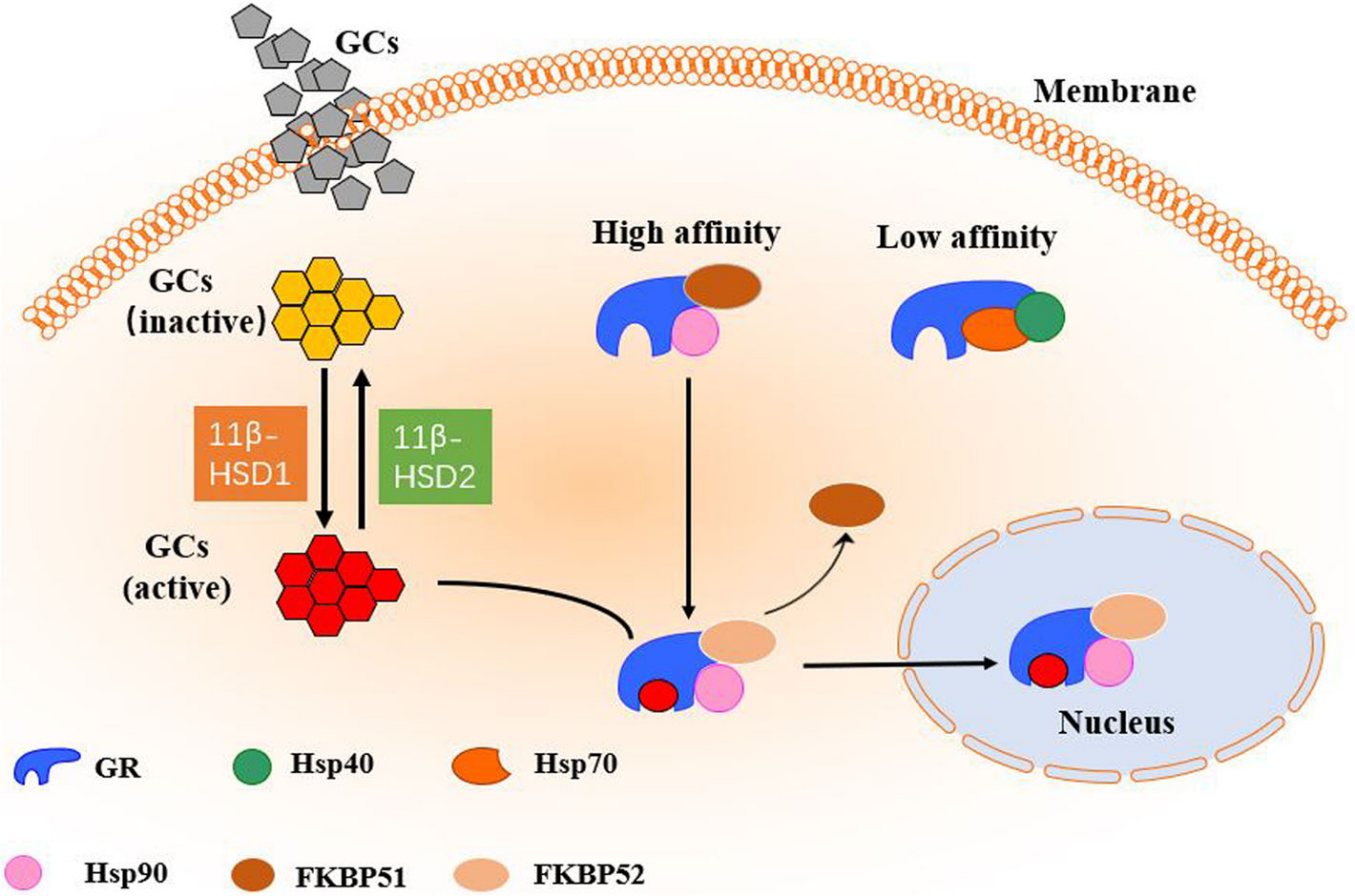
Glucocorticoid receptor activation. Upon entering into the cell, GCs are activated by 11β-HSD1 or occasionally inactivated by 11β-HSD2. The activated GCs bind to a cytoplasmic protein complex containing the GR and heat shock proteins. When complexed with Hsp90, the affinity of GR is increased, while when complexed with Hsp70 and Hsp40, its affinity is decreased. Once GR combines with the ligand, the chaperone protein FKBP51 is exchanged for FKBP52, allowing the complex to shuttle into the nucleus and interact with the chromatin

The GR, encoded by the *NR3C1* gene, is a member of the nuclear receptor superfamily of ligand-activated transcriptional factor. It mediates cellular effects of GCs and is widely expressed in almost all cells of the body [27, 28]. The GR consists of four major domains: the NH2-terminal transactivation domain (NTD) or activation function 1 (AF-1), DNA-binding domain (DBD), hinge region, and ligand-binding domain (LBD) [29]. The multiple domains of the GR are involved in ligand binding, DNA binding, and transcriptional regulation, which constitute the molecular basis of GC actions via the GR [8]. The GR controls transcription and modulates diverse physiological processes such as cell proliferation, differentiation, and apoptosis. Also, GR regulates gene transcription, either positively or negatively, by transactivation or transrepression, respectively [8]. After entering the cell, the ligand (GCs) binds to the receptor as part of large heterocomplexes (Fig. 1). This widely studied complex consists of several proteins, including heat shock proteins (e.g., HSP90 and HSP70), immunophilins such as FK506-binding proteins (FKBPs), CyP-40, P23, and perhaps few other proteins [30–32]. In the absence of ligand binding, the GR is primarily located in the cytoplasm, combined with immunophilins such as FKBP51 and FKBP52, heat shock/chaperone proteins (like Hsp70, Hsp90, and p23), and other proteins [31–34]. Hsp70 identifies newly synthesized GR molecules and binds to the LBD of GR [35, 36]. Another essential protein, co-chaperone protein Hsp40, promotes the combination of Hsp70 with GR to form a complex which has a low binding affinity to GCs. This complex allows for combination with the Hsp90 dimer, which enhances the affinity of the GR to the ligand [37]. While entering the cell, the activity and bioavailability of GCs are controlled by 11β-hydroxysteroid dehydrogenases 1 and 2 (11β-HSD1, 11β-HSD2), which act in the opposite manner [38]. The 11β-HSD1 converts cortisone (the inactive hormone form) to cortisol (the active hormone form), while 11β-HSD2 oxidizes cortisol to cortisone. The binding of GCs to the LBD of GR activates GR as a result of the complex being disassembled. Subsequently, the “activated” receptor enters the nucleus, where it interacts with critical sites of the regulated genes.

**Fig. 2:**
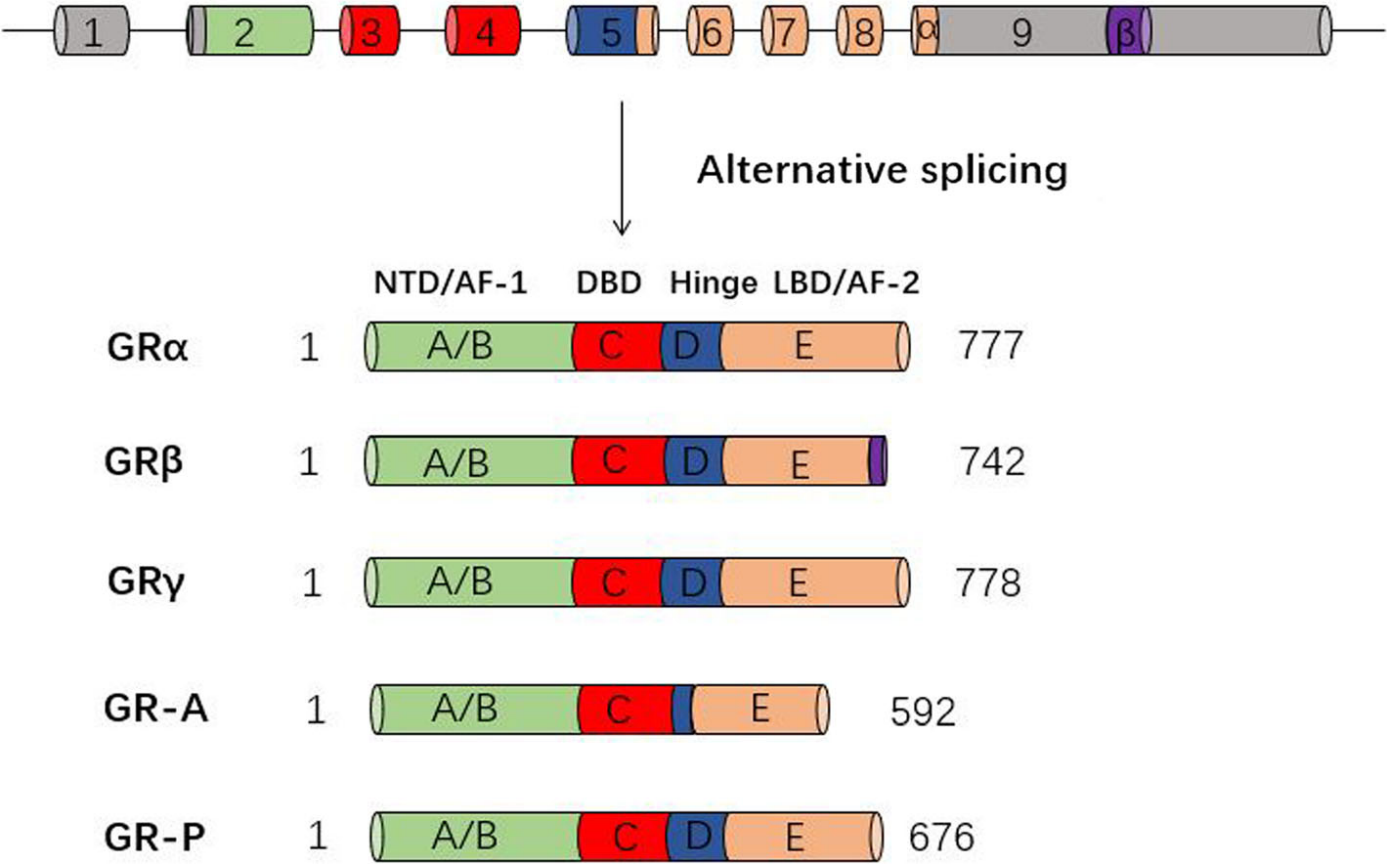
Domain structure of hGR splice variants and isoforms. The primary transcript of hGR consists of nine exons. Exon 1 constitutes the 5′-untranslated region (5′UTR), whereas the protein-coding region is made up of exons 2 to 9. In this process, the A/B-domain or NH2-terminal transactivation domain (NTD) containing the ligand-independent activation function 1 (AF-1) is encoded primarily by exon 2, the C-domain with the DNA-binding domain (DBD) is encoded by exons 3 and 4, and the D-domain or hinge region involved in nuclear localization is encoded by exon 5, while the other exons (up to exon 9) encode the E-domain including the ligand-binding domain (LBD) and a nuclear localization signal, which participates in the dimerization and Hsp90 binding

Alternative splicing of the pre-mRNA of the human glucocorticoid receptor (hGR) produces the GRα, GRβ, GRγ, GR-A, and GR-P isoforms [39–42] (Fig. 2). Isoforms GRα and GRβ are generated by alternative splicing of the GR transcript at exon 9 [43] and are 777 and 742 amino acids in length, respectively. Furthermore, isoforms GRα and GRβ are highly homologous in structure with different carboxyl termini. The GRα carboxy-terminus forms the LBD and is composed of 50 amino acids, while GRβ has only 15 carboxy-terminal amino acids and thus cannot bind GCs [44]. The GRα isoform is the most abundant and “classical” GR protein, while the GRβ isoform can regulate the expression of many genes that are not sensitive to regulation by GRα [45–47]. Being a ligand-dependent transcription factor, the binding of GRα to GCs can activate specific glucocorticoid response element (GRE) and regulate the expression of glucocorticoid-responsive genes. In contrast, hGRβ plays a dominant-negative role in the transcriptional activity of hGRα by occupying GRE target sites in a dose-dependent manner [39] and may contribute to GC resistance [48]. Real-time PCR and Western blot analysis results by YUN S P et al. showed that treatment with DEX (10^−6^ M) increases the expression level of hGRα mRNA and protein, but does not change hGRβ expression [49]. The GRγ isoform is produced by the insertion of an additional arginine between the two zinc fingers of the DBD of the GR [50]. Increased in vitro expression of GRγ is associated with GC resistance in initial acute lymphoblastic leukemia [51]. Furthermore, the GRγ isoform regulates genes that are not regulated by GRα, as revealed by expression profiling with GRγ overexpression [52]. In contrast to GRγ, the GR-A and GR-P isoforms are prevalent in some types of cancers, such as myeloma and leukemia, and cannot bind GCs due to exon deletions in their LBD [53, 54].

## Dose-dependent effect of GCs on the proliferation, osteogenesis, and adipogenesis of MSCs

Physiological levels of GCs stimulate osteoblast proliferation and promote osteogenic differentiation of MSCs. In contrast, pharmacological doses of GCs can not only induce apoptosis of osteoblasts and osteocytes but also reduce proliferation and inhibit the differentiation of osteoprogenitor cells. These effects of GCs are mediated by the GR [8]. It was shown that lower concentrations (10^−9^ M) of DEX promote the proliferation of rat BM-MSCs, whereas higher levels (10^−7^ and 10^−6^ M) inhibit proliferation [55]. Treatment of human BM-MSCs with DEX showed that DEX at the concentration of 10^−8^ M positively regulates the transcription of genes involved in cell proliferation [56]. The inhibitory effect of excess GC on the proliferation of osteoblastic lineage cells leads to GC-induced osteoporosis (GIO). Therefore, GCs have an impact on different mitogenic signaling pathways and attenuate the progression of the osteoblast cell cycle in a developmental stage-specific manner [57]. The Wnt signaling pathway can prevent apoptosis of osteoblasts and accelerate osteoblast cell cycle progression and thereby increase proliferation [58]. In murine osteoblastic MC3T3-E1 cells, the inhibition of cell cycle progression by DEX [59] is partly due to abnormal GR activation and subsequent p53 activation [60]. In MC3T3-E1 cells also, attenuation of proliferation is a prerequisite for osteoblast differentiation [59].

**Fig. 3:**
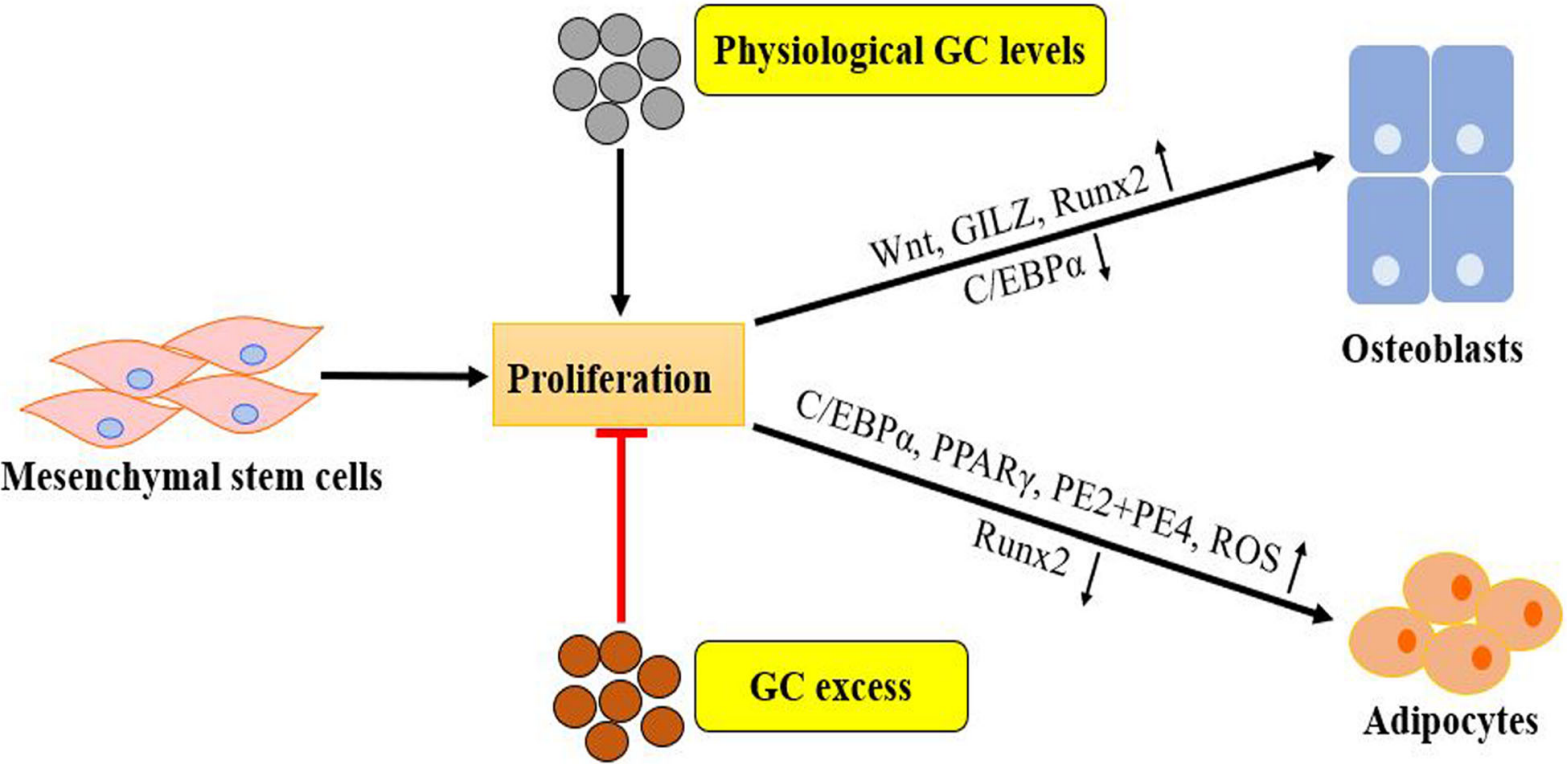
Effects of physiological and pharmacological concentrations of GCs on MSCs lineage commitment: excess GC inhibits MSC proliferation and shifts the MSC differentiation commitment toward adipogenic differentiation at the expense of osteogenesis by upregulating specific adipogenic transcription factors (PPARγ, C/EBPα) and prostaglandin receptors (PE2+PE4) and ROS or by inhibiting osteoblastogenic inducers (downregulation of Runx2). On the other hand, physiological GC levels can stimulate MSC proliferation and promote osteogenic differentiation of MSCs under certain conditions. Canonical Wnt signaling and GILZ might be implicated in this process

Besides, physiological levels of GCs play a positive regulatory role in osteoblast differentiation and bone formation, both in vitro and in vivo [61, 62]. A proper concentration of DEX (up to 10^−9^–10^−8^ M) is needed to trigger differentiation in human osteoblasts in a specific time-window, partly because it can increase the activity of alkaline phosphatase (ALP) during the early stages of osteoblast development [63]. The Wnt signaling pathway plays an essential role in the lineage commitment of MSCs from an adipogenic lineage into an osteoblastic lineage. The secreted frizzled-related protein 1, an inhibitor of the Wnt pathway, is downregulated by physiological concentrations of GCs [64]. Therefore, basal concentrations of GCs are crucial for the release of Wnt proteins, which then activates the canonical Wnt pathway in a paracrine manner (Fig. 3). Subsequently, β-catenin accumulates in the nucleus, and expression of the critical transcription factor Runx2 for osteoblast differentiation is promoted [8].

The use of excessive GC such as DEX suppresses osteogenic differentiation of MSCs and bone formation [16, 65] and shifts the lineage commitment of MSCs from the osteoblastic lineage to the adipocyte lineage [16, 66]. When the concentration of DEX is higher than 10^−8^ M, the osteogenic differentiation capacity of MSCs can be attenuated. It was indicated that C3H10T1/2 cells treated with high-dose DEX (10^−6^–10^−7^ M) have higher adipogenic differentiation potential, whereas low-dose DEX (10^−8^–10^−9^ M) cannot stimulate this potential [16]. The mechanisms underlying the effects of excess GCs or pharmacological dosages of GCs through the GR on osteogenic and adipogenic differentiation of MSCs are discussed in Table 1.

**Table 1:** Factors involved in the regulation of osteogenesis and adipogenesis by excess GC

Factors	Species	Cell type	In vitro*/*in vivo study	Molecular mechanism	Ref(s)
Signaling pathways					
Wnt/β-catenin signaling	Human Rat, mice	Osteoblasts BM-MSCs MC3T3-E1 cells	In vitro In vitro*,* in vivo	Enhance Wnt signaling pathway inhibitors (such as DKK1, SFRPs, Wif1, and sclerostin); downregulate Wnt7b and Wnt10	[67–72]
TGFβ/BMP superfamily	Mice	MC3T3-E1 cells	In vitro	Suppress BMP2; enhance BMP2 antagonists (follistatin and Dan)	[73, 74]
Notch signaling	Mice	MC3T3-E1 cells	In vitro In vivo	Enhance Notch1 and Notch 2; downregulate Notch target genes (such as Hey1, Hey2, and HeyL)	[75, 76]
Transcription factors/post-transcriptional regulators					
Runx2	Rat	Osteoblast BM-MSCs	In vitro In vitro	Inhibit Runx2; over-expression of Runx2 antagonizes GC-induced adipogenesis	[77–79]
PPARγ and C/EBPs	Mice Human	BM-MSCs 3T3-L1 preadipocytes BM-MSCs	In vitro In vitro In vitro	Enhance PPARγ and C/EBP members (C/EBPα, β, and δ); Downregulate C/EBPβ in the later stage of the adipogenesis process	[16, 80–82]
TAZ	Rat Mice	BM-MSCs 3T3-L1 preadipocytes	In vitro In vitro	Inhibit TAZ and ALP; overexpression of TAZ suppresses adipogenesis and promotes the trans-differentiation of preadipocytes into osteocytes	[83, 84]
GILZ	Mice Mice	BM-MSCs 3T3-L1 preadipocytes	In vitro In vivo	Activate GILZ; overexpression of GILZ enhances osteogenesis, inhibits adipogenesis by inhibiting PPARγ2 and C/EBPα, and GILZ inhibits PPAR-γ2 mediated by C/EBP-δ	[85–88]
Prostaglandin E2	Human	BM-MSCs	In vitro	Induce prostaglandin receptors to inhibit osteogenesis and enhance adipogenesis	[89]
MicroRNAs’ detailed information is shown in Table 2					
Long non-coding RNAs	Rat Human	BM-MSCs BM-MSCs	In vitro In vitro	Downregulate lncRNA TCONS_00041960 and lncRNA RP11-154D6; lncRNA TCONS_00041960 enhances osteogenesis and inhibits adipogenesis by targeting miR-204-5p and miR125a-3p	[96, 97]
Other regulators					
Oxidative stress	Mice Rat	Osteoblasts Osteoblasts	In vitro In vitro	Increase ROS to decrease Cbfa1 expression; N-acetylcysteine mitigates the detrimental effects of GC-induced oxidative stress	[98, 99]
FilGAP–FLNA	Human	BM-MSCs	In vitro	Antagonize mechanical forces; FLNA and c-Tubulin play an important role in mechanical regulation during osteogenesis and adipogenesis	[100, 101]

Factors, signaling pathways, transcription factors and post-transcriptional regulators, and other regulators, involved in the regulation of osteogenesis and adipogenesis by GC excess; Species, species involved in study; Cell type, cell type involved in the study; In vitro*/*In vivo Study, study performed in vitro or in vivo; Molecular mechanism, molecular mechanism involved in the regulation of osteogenesis or adipogenesis by excess GC; References, references related to the study in this table

## Major signaling pathways involved in adipo-osteogenic differentiation of MSCs by excess GCs

The differentiation commitment of MSCs is regulated by several signaling pathways which can promote MSCs to differentiate into osteoblasts, adipocytes, chondrocytes, and stromal cells. Different signaling molecules can also regulate the differentiation commitment of MSCs via a variety of signaling pathways. The various GR-mediated signaling pathways that affect osteogenic or adipogenic differentiation of MSCs are represented in Table 1.

### Wnt/β-catenin signaling

As an evolutionarily conserved signaling pathway, the Wnt signaling pathway has been widely studied and is crucial for renewal, proliferation, and differentiation of stem cells during embryonic development and adult tissue homeostasis [102]. The binding of Wnt ligands, such as secreted frizzled-related proteins (SFRPs), to the frizzled receptor and low-density lipoprotein receptor-related protein 5 or 6 (LRP5 or LRP6) can stabilize β-catenin by preventing its phosphorylation and activate the Wnt pathway [103]. Accumulating evidence suggests that the activated Wnt signaling promotes osteogenic differentiation [104] and inhibits adipogenic differentiation of MSCs [105]. Glucocorticoids (GCs) affect Wnt signaling through different mechanisms. On the one hand, high doses of GCs attenuate osteoblast differentiation in mice in vitro by reducing the expression of Wnt7b and Wnt10. On the other hand, high GC doses further enhance the expression of inhibitors of the Wnt signaling pathway, such as dickkopf-1 (DKK1) [67, 68], SFRPs [69, 70], Wnt inhibitory factor-1(Wif1), and sclerostin [71]. Accordingly, knocking down DKK1 attenuates the negative effects of GCs on bone mass in rats [72]. Moreover, serum from children who have been treated with high GC doses for long-term express elevated levels of DKK1 [106].

### TGFβ/BMP superfamily

Transforming growth factor-beta (TGFβ)/bone morphogenic protein (BMP) signaling pathway plays dual roles in regulating osteogenic and adipogenic differentiation of MSCs [107]. The TGF-β superfamily only exists in mammals and can be divided into three closely related subtypes: TGF-β1–TGF-β3, and BMPs. The BMPs, which represent the largest subgroup of this superfamily, belong to the TGFβ1 family [108]. By binding to transmembrane serine-threonine kinase receptors (termed type I and type II), TGFβ/BMP ligands activate the canonical Smad-dependent signaling pathways (TGFβ/BMP ligands, receptors, Smads) and the non-canonical Smad-independent signaling pathway such as p38 mitogen-activated protein kinase (MAPK) pathway [109]. Following TGFβ/BMP induction, either the Smad or p38 MAPK pathway can regulate the expression of the runt-related gene 2 (Runx2) [108] and the peroxisome proliferator-activated receptor-gamma (PPARγ) [107]. Also, the osteogenic or adipogenic differentiation of MSCs is directly controlled by alteration in the expression levels of lineage-specific transcription factors such as Runx2 or PPARγ. In vitro, treatment with BMP-2, a key regulator of osteoblast differentiation, enhances osteoblast differentiation by increasing the expression of *Runx2* gene and ALP in several types of MSCs, such as human marrow stromal cell line [110] and human adipose-derived stem cells (ASCs) [111]. Glucocorticoids (GCs) suppress the expression of BMP2 gene, which can antagonize the GC-induced inhibitory effects of differentiation depending on the differentiation stage in osteoblastic MC3T3-E1 cells culture model [73]. In MC3T3-E1 cells also, GCs increase the mRNA expression of follistatin and Dan, which antagonize BMP2 [74].

### Notch signaling

Notch signaling functions as a molecular gate and is closely correlated with binary cell fate decisions during embryonic development and homeostatic maintenance of adults [112–115]. Mammals have five classic Notch ligands (Jagged-1 and Jagged-2, Delta-like 1, 3, and 4) and four transmembrane notch receptors (Notch1–4) [116]. Notch ligands are single-pass transmembrane proteins and play the dual roles of activating and repressing Notch signaling [117]. The trans-interaction of a Notch ligand-receptor results in receptor activation, whereas a cis-interaction in the same cell inhibits Notch signaling [118]. The Notch signaling pathway is activated by an interaction between a membranous Notch receptor and the membrane-bound ligand of adjacent cells, which lead to proteolytic cleavage of the Notch receptor. After that, the Notch intracellular domain is released and translocates into the nucleus to regulate the expression of related genes. The role of Notch signaling in the lineage decision of MSCs is essential and complicated. As revealed in the 3T3-L1 pre-adipocyte model, for instance, Notch signaling plays dual roles in adipogenic differentiation [119]. Similar to the dual roles in adipogenesis, the interaction between Notch1 and BMP-2, a TGFβ ligand, enhances osteogenic differentiation and ectopic bone formation of MC3T3-E1 cells in vivo [120]. However, the overexpression of Notch1 inhibits osteogenesis of mice BM ST-2 cells by suppressing Wnt/β-catenin [121]. The findings of a genome-wide association study indicate that the *JAG1* gene, which encodes the Notch ligand Jagged-1, is associated with bone mineral density and osteoporotic fractures [122]. Although GCs can inhibit osteoblast differentiation by stimulating mRNA expression of Notch1 and Notch 2, GCs do not regulate the expression of Notch ligands in MC3T3-E1 cells [75]. Additionally, in vivo administration of GCs inhibits the expression of Notch target genes such as *Hey1*, *Hey2*, and *HeyL* in osteoblasts [76].

## Transcription factors and post-transcriptional regulators involved in adipo-osteogenic differentiation of MSCs by excess GCs

The lineage commitment of MSCs is also modulated by transcription factors or post-transcriptional regulators, which can directly or indirectly act on target genes of various signaling pathways, thus initiating and promoting osteogenic or adipogenic differentiation of MSCs. Glucocorticoids (GCs) can positively or negatively regulate the expression of multiple transcription factors (e.g., PPARγ, Runx2) and post-transcriptional regulators (e.g., miRNAs, lncRNAs), as shown in Table 1, thereby affecting the differentiation of MSCs into osteoblasts or adipocytes (Fig. 3).

### Runx2

Runt-related transcription factor 2 (Runx2/Cbfa1) is an essential differentiation factor and a key regulator of osteoblast differentiation in mammals [123–125]. The critical function of Runx2 in osteogenesis was elucidated in mice with a homozygous Runx2 mutation. The Runx2^−/−^ mice died soon after birth and exhibited an incompletely ossified skeleton [126]. According to a study, GCs can rapidly inhibit the expression of functional Runx2 in nuclear extracts from the GC-treated primary osteoblast cultures of fetal rat [77]. In another study, DEX-activated GR exerts various degrees of antagonistic effects on Runx2 during osteoblast differentiation in cultures of BM-derived ST2 pluripotent mesenchymal cells [78]. Besides, the over-expression of Runx2 can antagonize the effect of GC-induced adipogenic differentiation on primary cultured rat BM-MSCs [79].

### PPARγ and C/EBPs

The nuclear receptor peroxisome proliferator-activated receptor-gamma (PPARγ) and CCAAT/enhancer-binding proteins (C/EBPs) are transcription factors involved in the regulation of adipogenesis [80, 127–129]. Glucocorticoids (GCs) induce the expression of PPARγ and C/EBP members (C/EBPα, β, and δ), leading to enhanced adipogenic differentiation and decreased osteoblastogenesis in BM-MSCs [16, 81, 82]. Meanwhile, the binding of DEX to endocellular GR can activate the expression of C/EBPδ [80]. The expression of PPARγ and C/EBPα is upregulated during the entire adipogenesis process of 3T3-L1 preadipocytes, while the expression of C/EBPβ is downregulated in the later stage of the process [80].

### TAZ

The transcriptional co-activator with PDZ binding motif (TAZ) is a transcriptional co-activator that regulates the differentiation of MSCs into osteoblasts or adipocytes [125]. The expression of TAZ promotes osteogenic differentiation of MSCs by coactivating *Runx2*-dependent gene transcription and blocks adipogenic differentiation of MSCs by inhibiting *PPARγ*-dependent gene transcription [130–132]. The expression of TAZ and ALP were enhanced by treatment with DEX at a concentration of 10^−8^ M during osteoblastic differentiation of rat BM-MSCs, whereas a higher concentration (10^−7^ M) of DEX strongly suppressed the expression of TAZ and ALP [83]. The expression of TAZ is inhibited by a high concentration of DEX at the transcriptional level during the differentiation of 3T3-L1 preadipocytes into mature adipocytes. Furthermore, overexpression of TAZ suppresses adipogenesis and trans-differentiates 3T3-L1 preadipocytes into osteocytes [84].

### Glucocorticoid-induced leucine zipper

G*lucocorticoid-induced leucine zipper* (*GILZ*), one of the GC-induced genes [133], belongs to the leucine zipper protein family and mediates the anti-inflammatory effect of GCs [134–136]. Classic GCs activate GR and transactivate the anti-inflammatory protein GILZ [85]. Overexpression of GILZ promotes osteogenic differentiation of mice BM-MSCs [86] and enhances bone acquisition in GILZ transgenic mice [87]. In contrast, inhibition of the expression of PPARγ2 and C/EBPα reduces the adipogenic differentiation capacity of mice BM-MSCs over-expressing GILZ [86]. Another study reported similar results that over-expression of GILZ blocks the differentiation of 3T3-L1 preadipocytes into adipocytes, and GILZ inhibits the expression of PPAR-γ2 mediated by C/EBP-δ [88].

### Prostaglandin E2

Prostaglandin E2 (PGE2), a potent lipid mediator and a pro-inflammatory mediator, plays a vital role in skeletal metabolism and bone cell differentiation [137, 138]. Due to the induction of prostaglandin receptors (mainly PE2 and PE4 receptor subtypes) in human BM-MSCs, DEX enables these cells to respond to PGE2 which then shift the lineage commitment of BM-MSCs from osteogenic to adipogenic differentiation [89].

### MicroRNAs

MicroRNAs (MiRNAs) are 18–25-nt long small non-coding RNAs that regulate gene expression at the post-transcriptional level by binding to the 3′ untranslated region (3′UTR) of particular target mRNAs [139, 140]. The binding of miRNAs to target mRNAs results in the degradation of target mRNAs or inhibition of mRNA translation, hence gene silencing [141]. In human BM-MSCs, the silencing of either *Dicer* or *Drosha*, two key enzymes involved in the biogenesis of miRNA, inhibits differentiation of MSCs into osteoblasts and adipocytes [142]. Accumulating evidence reveals that miRNAs are essential regulators of MSC differentiation and fate decisions [143] (Table 2). For example, miR-29a promotes osteogenic differentiation of human MSCs in vitro by targeting HDAC4, a negative regulator of osteoblast differentiation [90]. In the human mesenchymal precursor cell line hFOB1.19, canonical Wnt signaling promotes transcription of miR-29a, which can potentiate Wnt signaling by inhibiting key Wnt signaling antagonists such as Dkk1 and Kremen2 [91]. The microRNA-29a promotes osteogenic differentiation by inhibiting GC-induced blockage of Wnt signaling in GC-treated rats, and thus, modulating miR-29a signaling is a promising strategy for alleviating GC-induced bone loss [92]. Using miRNA microarrays and RT-PCR, Li et al. [93] identified nine upregulated miRNAs and seven downregulated miRNAs involved in osteogenic differentiation of DEX-induced human BM-MSCs. Target genes of the upregulated DEX-induced miRNAs include RUNX2, SMAD1, SMAD5, and BMPR2, which promote osteogenic differentiation of human BM-MSCs. Similarly, target genes of the downregulated miRNAs induced by DEX treatment include Sox4, BMP3, HDAC4, and TGF-β1, and these inhibit osteogenic differentiation. In another study, miR-216a can reverse the inhibitory effect of DEX on osteogenic differentiation of human adipose-derived MSCs in vitro and enhance ectopic bone formation in vivo, by suppressing the expression of c-Cbl [94]. In contrast, miR-708 can promote steroid-induced osteonecrosis of the femoral head (SONFH) and inhibit the osteogenic differentiation of human BM-MSCs in vitro by targeting SMAD3 [95].

**Table 2:** MiRNAs involved in osteogenesis and adipogenesis of GC-treated MSCs

MiRNAs	Species	In vitro*/*in vivo study	Expression	Functions	Target genes	Ref(s)
MiR-29a	Human, Rat Rat	In vitro In vivo	Down	Osteogenesis	HDAC4	[90–92]
MiR-27a	Human	In vitro	Up	Osteogenesis and inhibition of adipogenesis	RUNX1, SMAD5, SATB2, LRP6, FOXO1	[93]
MiR-22	Human	In vitro	Up	Osteogenesis and inhibition of adipogenesis	SMAD4, SATB2, HDAC6	[93]
MiR-23a	Human	In vitro	Up	Osteogenesis	SATB1, STAT5B, TMEM135, FGF2, NFIB, SMAD5, IHH, RUNX2	[93]
MiR-221	Human	In vitro	Up	Osteogenesis	Wnt1, Sox4	[93]
MiR-26a	Human	In vitro	Up	Osteogenesis	SMAD1, Wnt5α, SMAD4, TMEM135	[93]
MiR-130a	Human	In vitro	Up	Osteogenesis	SMAD5, SMAD4, BMPR1B, BMPR2	[93]
MiR-199a-5p	Human	In vitro	Up	Osteogenesis	TMEM135, Sox4	[93]
MiR-196a	Human	In vitro	Up	Osteogenesis and inhibition of adipogenesis	COL1A1, COL1A2, COL3A1, FOXO1	[93]
MiR-155	Human	In vitro	Up	Osteogenesis	SATB2	[93]
MiR-21	Human	In vitro	Down	Inhibit of osteogenesis	SMAD7, Sox2, TGFBR2	[93]
MiR-140-3p	Human	In vitro	Down	Inhibit of osteogenesis	Acvr2b, CBL, HDAC4	[93]
MiR-214	Human	In vitro	Down	Inhibit of osteogenesis and adipogenesis	CBL, GSK3β	[93]
MiR-744	Human	In vitro	Down	Inhibit of osteogenesis	TGFβ1	[93]
MiR-320a	Human	In vitro	Down	Inhibit of osteogenesis	CBL, BMP3, HDAC4, Sox4, Acvr2b, TGFBR2	[93]
MiR-320b	Human	In vitro	Down	Inhibit of osteogenesis	CBL, BMP3, HDAC4, Sox4, Acvr2b, TGFBR2	[93]
MiR-320c	Human	In vitro	Down	Inhibit of osteogenesis	CBL, BMP3, HDAC4, Sox4, Acvr2b, TGFBR2	[93]
MiR-216a	Human Mice	In vitro In vivo	Up	Osteogenesis	c-Cbl	[94]
MiR-708	Human	In vitro	Up	Inhibition of osteogenesis	SMAD3	[95]

Species, species involved in study; In vitro*/*in vivo study, study performed in vitro or in vivo; Expression, expression of miRNAs during osteogenic or adipogenic differentiation of GC-treated MSCs: Up, upregulation, Down, downregulation; Functions, functions of miRNAs during osteogenic or adipogenic differentiation of GC-treated MSCs; Target genes, target genes determined or predicted by the study; References, references related to the study in this table

### Long non-coding RNAs

Long non-coding RNAs (lncRNAs) are a class of non-protein coding RNAs with a length of more than 200 nucleotides [144]. Mounting evidence has demonstrated that lncRNAs participate in diverse critical biological processes such as growth and differentiation of various cell types [145, 146]. Similar to microRNAs, lncRNAs play a critical role in the adipo-osteogenic lineage commitment of MSCs [147–149]. For instance, the lncRNA TCONS_00041960 promotes osteogenic differentiation and suppresses adipogenic differentiation of GC-treated rat BM-MSCs, by directly sponging miR-204-5p and miR125a-3p [96]. In another study, the expression of the lncRNA RP11-154D6 was reduced in human BM-MSCs of patients with SONFH, and its overexpression enhanced osteogenic differentiation while inhibiting adipogenic differentiation [97]. However, more lncRNAs involved in osteogenesis and adipogenesis via GR need to be identified and studied.

## Other regulators controlling the balance between osteogenesis and adipogenesis of MSCs

### Oxidative stress

Oxidative stress represents an unbalanced situation in which the production of reactive oxygen species (ROS) overwhelms the cell’s antioxidant systems, causing tissue damage. Mesenchymal stem cells (MSCs) typically have low levels of intracellular ROS and high levels of glutathione, an important antioxidant [150]. As living organisms age, ROS and oxidative stress levels increase, and the two play important roles in age-related bone loss and conversion of adipo-osteogenic differentiation through Wnt, PPARγ, and Forkhead box O (FoxO) [151–153]. Increasing evidence indicates that changes in metabolic processes, such as mitochondrial metabolism and oxidative stress, affect osteogenic and adipogenic differentiation of MSCs [154, 155]. For example, increased ROS generation inhibits the proliferation and osteogenic differentiation of rat BM-MSCs [156]. Low levels of ROS facilitate osteogenesis, whereas high levels of ROS promote adipogenic differentiation of MSCs [150]. Besides, the administration of GCs to mice increases ROS in bone, and GCs suppress Wnt-induced proliferation and osteoblast differentiation of the mice osteoblastic cell line [98] (Fig. 3). Oxidative stress-induced by DEX (10^−7^, 10^−6^, 10^−5^ M) decreases the Cbfa1 mRNA expression and inhibits differentiation of rat osteoblasts, whereas the antioxidant *N*-acetylcysteine (NAC) can mitigate the damaging effects of DEX-induced oxidative stress [99].

### FilGAP-filamin A

Darling et al. found that human MSC-derived osteoblasts, chondrocytes, and adipocytes exhibit distinct mechanical properties, with osteoblasts being stiffer than chondrocytes, and both being stiffer than adipocytes [157]. This exciting phenomenon demonstrates that cellular differentiation and mechanical properties are correlated. The study also showed that the presence of an appropriate mechanical stimulus could promote osteogenic differentiation and inhibit adipogenic differentiation of rat BM-MSCs [158]. *Filamin A* (*FLNA*), a cytoskeletal gene, is a member of the high-molecular-mass cytoskeletal family of proteins that cross-link actin filaments and link actin networks to cell membranes [159–161]. The *FLNA* gene is induced by mechanical forces applied through β1 integrins [162, 163]. The Rac GTPase-activating protein (GAP) FilGAP regulates cellular activity such as actin assembly [164] and targets sites of FLNA force transfer [165]. Concomitant upregulation of FLNA and c-Tubulin with cellular elastic modulus indicated that FLNA and c-Tubulin play an essential role in mechanical regulation during adipogenic and osteogenic differentiation of human BM-MSCs [100]. On the other hand, mechanical stimuli protect against apoptosis of osteocytes and osteoblasts by activating the focal adhesion kinase (FAK) and extracellular signal-regulated kinases (ERKs) [166]. Meanwhile, GCs antagonize these survival signals by the activation of pro-apoptotic kinases Pyk2 and c-Jun N-terminal kinase (JNK) [167]. An antagonistic effect, therefore, exists between mechanical forces and GC, governed by FAK/Pyk2 signaling that acts on the Wnt pathway and regulates apoptosis of osteoblasts and osteocytes [101]. The interaction between FilGAP and FLNA in response to mechanical stimuli plays a mechano-protective role in preventing cells from force-induced apoptosis [165].

## Conclusion and perspective

The findings of various studies have demonstrated that GCs can exert a significant effect via several signaling pathways on the shift between osteogenic and adipogenic differentiation of MSCs. These signaling pathways eventually converge at several transcription factors. For osteogenic differentiation, the pathways converge at Runx2 and TAZ and PPARγ and C/EBPs for adipogenic differentiation of MSCs, participating in a series of tightly controlled transcription events [168, 169]. Other transcription factors and post-transcriptional regulators, such as GILZ, PGE2, miRNAs, and lncRNAs, also affect the delicate balance between osteogenic and adipogenic differentiation of MSCs. Besides, other regulators, such as oxidative stress and FilGAP-filamin A, are also involved in osteogenesis and adipogenesis of MSCs.

Based on its function and role in osteoblastogenesis and adipogenesis of MSCs, GR may modify MSC lineage decisions. To reduce the adverse side effects of GC on the skeletal system, such as bone loss, fractures, and osteonecrosis, new and effective therapies that enhance bone formation by stimulating osteoblast differentiation should be developed. Notably, selective GR modulators such as compound A (CpdA) have been proposed. CpdA, a nonsteroidal anti-inflammatory compound, does not induce GR dimerization or transcriptional activation nor does it interfere with osteoblast differentiation in vitro and in vivo [170]. Furthermore, this GR-modulating agent has a bone-sparing effect in mice [171]. We anticipate that leveraging on cell-based screening methods may lead to the development of additional bone-sparing GR ligands. Secondly, agents that activate bone cell-protective effects by modulating GR-mediated pathways could be effective alternatives. Sclerostin, secreted by osteocytes, is an inhibitor of the Wnt signaling which attenuates osteoblast function and differentiation. Excessive GC has been shown to stimulate sclerostin secretion from osteocytes. Neutralizing antibodies against sclerostin, such as romosozumab, can promote Wnt signaling and improve osteoblast function [172]. Elsewhere, it has been reported that miR-29a can suppress the side effects of excessive GCs on osteoblast differentiation in vitro and restore Wnt signaling by regulating β-catenin acetylation [173]. To prevent the production of excessive ROS levels following GC administration and redirect the MSC fates, effective pharmaceutical strategies such as the use of antioxidants, extracts from natural products, and gene modifications have been proposed to potentiate the therapeutic efficacy of MSCs [174]. In addition, tissue-specific or cell type-specific delivery systems for GCs may minimize the systemic distribution of pharmacological GCs and thus reduce the adverse effects of GCs on skeletal system.

BM-MSCs have been extensively studied and applied in tissue regeneration and stem cell-based therapies, but the numbers of MSCs in BM are relatively low [175, 176]. In contrast, adipose-derived stem cells (ASCs) are easily obtainable and expandable to provide sufficient numbers for therapeutic applications. Megestrol acetate (MA) imparts mitogenic effects on ASCs and increases the proliferation of ASCs via GR phosphorylation [177]. Future studies should compare in vitro results with in vivo preclinical findings and establish stem cell banks based on the heterogeneity of MSC subpopulations. Furthermore, stem cell therapy based on different MSC subgroups with some advantages (e.g., osteogenesis, adipogenesis, chondrogenesis, angiogenesis, immunomodulation, and hematopoiesis support) should be applied to selectively treat various diseases. Although remarkable advancements in the field of stem cell therapy have expanded our understanding of the role played by GC on MSC phenotypes in vitro, the effects of GCs on mesenchymal precursor cells in vivo remains unknown. In vivo studies are, therefore, required to explore the mechanisms of GR on lineage decisions of MSCs to generate ideas that can be used to develop novel therapies that suppress the negative effects of GCs on the skeletal system, and to improve the clinical application of MSCs in tissue engineering and regenerative medicine.

## Data Availability

Not applicable.

## Abbreviations

AF-1: Activation function 1
ALP: Alkaline phosphatase
ASCs: Adipose-derived stem cells
BM: Bone marrow
BM-MSCs: Bone marrow-derived MSCs
BMP: Bone morphogenic protein
C/EBPs: CCAAT/enhancer-binding proteins
DBD: DNA-binding domain
DEX: Dexamethasone
DKK1: Dickkopf-1
ERKs: Extracellular signals regulated kinases
FAK: Focal adhesion kinase
FKBPs: FK506-binding proteins
FLNA: Filamin A
FoxO: Forkhead box O
GAP: GTPase-activating protein
GC(s): Glucocorticoid(s)
GILZ: Glucocorticoid-induced leucine zipper
GIO: GC-induced osteoporosis
GR: Glucocorticoid receptor
GRE: Glucocorticoid response element
HSD: Hydroxysteroid dehydrogenases
HSP: Heat shock protein
JNK: Jun N-terminal kinase
LBD: Ligand-binding domain
LncRNAs: Long non-coding RNAs
LRP: Low-density lipoprotein receptor-related protein
MA: Megestrol acetate
MAPK: Mitogen-activated protein kinase
MiRNAs: MicroRNAs
MSCs: Mesenchymal stem cells
NAC: *N*-Acetylcysteine
NTD: NH2-terminal transactivation domain
OA: Osteoarthritis
PGE: Prostaglandin E
PPARγ: Peroxisome proliferator-activated receptor-gamma
ROS: Reactive oxygen species
Runx2: Runt-related transcription factor 2
SFRPs: Secreted frizzled-related proteins
SONFH: Steroid-induced osteonecrosis of the femoral head
SSCs: Skeletal stem cells
TGFβ: Transforming growth factor-beta
UTR: Untranslated region
Wif1: Wnt inhibitory factor-1
